# Impaired Overall Survival of Melanoma Patients Due to Antibiotic Use Prior to Immune Checkpoint Inhibitor Therapy: Systematic Review and Meta-Analysis

**DOI:** 10.3390/cancers17111872

**Published:** 2025-06-03

**Authors:** Thilo Gambichler, Sera S. Weyer-Fahlbusch, Jan Overbeck, Nessr Abu Rached, Jürgen C. Becker, Laura Susok

**Affiliations:** 1 Skin Cancer Center, Department of Dermatology, Ruhr-University Bochum, 44791 Bochum, Germany; nessr.aburached@klinikum-bochum.de (N.A.R.); laura.susok@klinikumdo.de (L.S.); 2 Department of Dermatology, Klinikum Dortmund gGmbH, University Witten/Herdecke, 44137 Dortmund, Germany; seraselina.weyer-fahlbusch@klinikumdo.de (S.S.W.-F.); jan.overbeck@uni-wh.de (J.O.); 3 Department of Dermatology, Christian Hospital Unna, 59423 Unna, Germany; 4 Translational Skin Cancer Research, DKTK Partner Site Essen/Düsseldorf, West German Cancer Center, Dermatology, University Duisburg-Essen, 45147 Essen, Germany; j.becker@dkfz-heidelberg.de; 5 German Cancer Research Center (DKFZ), 69120 Heidelberg, Germany

**Keywords:** antibiosis, infection, mortality, immunotherapy, nivolumab, pembrolizumab, ipilimumab

## Abstract

**Background:** The gut microbiome plays a pivotal role in shaping systemic immunity and modulating anti-tumor responses. Preclinical and clinical studies have shown that higher gut microbial diversity and the presence of specific commensal taxa correlate with improved responses to immune checkpoint inhibitors (ICI) in melanoma. Conversely, broad-spectrum antibiotics can induce dysbiosis, reducing T cell activation and cytokine production, and have been linked to diminished ICI efficacy in several cancer types. **Methods:** We conducted a systematic review and meta-analysis of seven retrospective cohorts (total n = 5213) comparing overall survival in cutaneous melanoma (CM) patients who did or did not receive systemic antibiotics within six weeks before ICI initiation. From each study, we extracted hazard ratios (HRs) for death, antibiotic-to-ICI interval, ICI regimen (PD-1 monotherapy vs. PD-1 + CTLA-4 combination), cohort size, and country. Pooled log-HRs were estimated under fixed-effect and random-effects (REML) models. Statistical heterogeneity was quantified by Cochran’s Q and I^2^ statistics, and τ^2^. We performed leave-one-out sensitivity analyses, generated a Baujat plot to identify influential studies, applied trim-and-fill to assess publication bias, and ran meta-regressions for regimen, antibiotic timing, sample size, and geography. **Results:** Under the fixed-effect model, antibiotic exposure corresponded to a pooled HR of 1.26 (95% CI 1.13–1.41; *p* < 0.001). The random-effects model yielded a pooled HR of 1.55 (95% CI 1.21–1.98; *p* = 0.0005) with substantial heterogeneity (Q = 25.1; I^2^ = 76%). Prediction intervals (0.78–3.06) underscored between-study variability. Leave-one-out analyses produced HRs from 1.50 to 1.75, confirming robustness, and the Baujat plot highlighted two cohorts as primary heterogeneity drivers. Trim-and-fill adjusted the HR to 1.46 (95% CI 1.08–1.97). In subgroup analyses, combination therapy studies (k = 4) showed a pooled HR of ~1.9 (I^2^ = 58%) versus ~1.3 (I^2^ = 79%) for monotherapy. Meta-regression attributed the largest variance to the regimen (R^2^ = 32%; β(monotherapy) = −0.35; *p* = 0.13). **Conclusions:** Pre-ICI antibiotic use in CM is consistently associated with a 26–55% increase in mortality risk, particularly with PD-1 + CTLA-4 combinations, reinforcing the mechanistic link between microbiome integrity and ICI success. Looking ahead, integrating prospective microbiome profiling into clinical trials will be critical to personalize ICI therapy, clarify causality, and identify microbial biomarkers for optimal treatment selection. Prospective, microbiome-integrated trials promise to refine melanoma immunotherapy by tailoring antibiotic stewardship and microbial interventions to enhance patient outcomes.

## Simple Summary

The gut microbiome influences how well melanoma patients respond to immune checkpoint inhibitors (ICI), and broad-spectrum antibiotics can disrupt this balance. We pooled data from seven retrospective cohorts (5213 patients) who either did or did not receive antibiotics within six weeks before starting ICI therapy. Across studies, patients who had taken antibiotics faced a 26–55% higher risk of death, with those on combination PD-1 plus CTLA-4 therapy experiencing the greatest impact (nearly two-fold increase). Although study differences, such as ICI regimen, timing, and geography, contributed to variability, the antibiotic-ICI association remained robust even after adjusting for potential publication bias. These findings highlight the need to preserve microbiome health through careful antibiotic use and underscore the importance of future trials that include detailed microbiome assessments to guide personalized ICI treatment in melanoma.

## 1. Introduction

Metastatic cutaneous melanoma (CM) has historically carried a poor prognosis, but the advent of immune checkpoint inhibitors (ICI) targeting PD-1 and CTLA-4 has dramatically improved survival outcomes. Despite these advances, only a subset of patients have achieved durable benefit, and conventional biomarkers, such as PD-L1 expression, tumor mutational burden, and circulating immune cell subsets, have been only partially successful in predicting response. Accordingly, there is a pressing need to identify additional host- and treatment-related factors that modulate ICI efficacy [1,2,3].

Emerging evidence implicates the gut microbiome as a critical modulator of anti-tumor immunity. Preclinical models demonstrate that specific bacterial taxa enhance T-cell priming and ICI responsiveness, whereas antibiotic-induced dysbiosis impairs these effects [4,5]. Clinical studies have correlated baseline microbial diversity with objective response rates and immune-related adverse events in CM and other solid tumors [6,7], suggesting that perturbations of the microbiome—particularly antibiotic use—could undermine the therapeutic benefit of ICIs.

Numerous observational and registry-based studies have examined the impact of antibiotics on ICI outcomes across a variety of malignancies, yet their methodologies and patient populations have differed widely [8,9,10,11,12,13,14,15,16,17,18,19,20]. A broad systematic review and meta-analysis in mixed solid tumors first reported that pericritical antibiotic exposure was linked to poorer ICI response and survival but included cancers ranging from lung to renal cell carcinoma [8,9]. Subsequent analyses specific to melanoma have varied in design: some leveraged large health insurance databases to capture elderly cohorts [12], while others conducted detailed chart reviews in single-center series [10,15]. Study definitions of “antibiotic exposure” have not been uniform; window lengths span from two weeks to three months before the start of ICI, and the antibiotics themselves range from narrow-spectrum to broad-spectrum agents [13,14]. In some reports, concomitant medications such as proton-pump inhibitors and steroids were co-analyzed and further complicated interpretation [18,19], whereas other cohorts focused solely on systemic antibacterial use [16,17]. A recent umbrella review highlighted these inconsistencies and called for standardized exposure definitions and rigorous adjustment for confounders [20]. Because estimates of the antibiotic–ICI relationship in CM have ranged from null to markedly adverse, we sought to aggregate and critically appraise these data through a comprehensive meta-analysis focused on CM. In order to reconcile these discrepancies and quantify the effect of pre-ICI antibiotics on overall survival (OS) in CM, we performed a systematic meta-analysis of published cohort studies. We sought to estimate the overall association between antibiotic exposure and mortality and to explore sources of between-study heterogeneity, such as antibiotic-to-ICI interval, ICI regimen, study size, and geographic setting, through subgroup analyses and meta-regression.

## 2. Materials and Methods

### 2.1. Protocol

**Figure 1:**
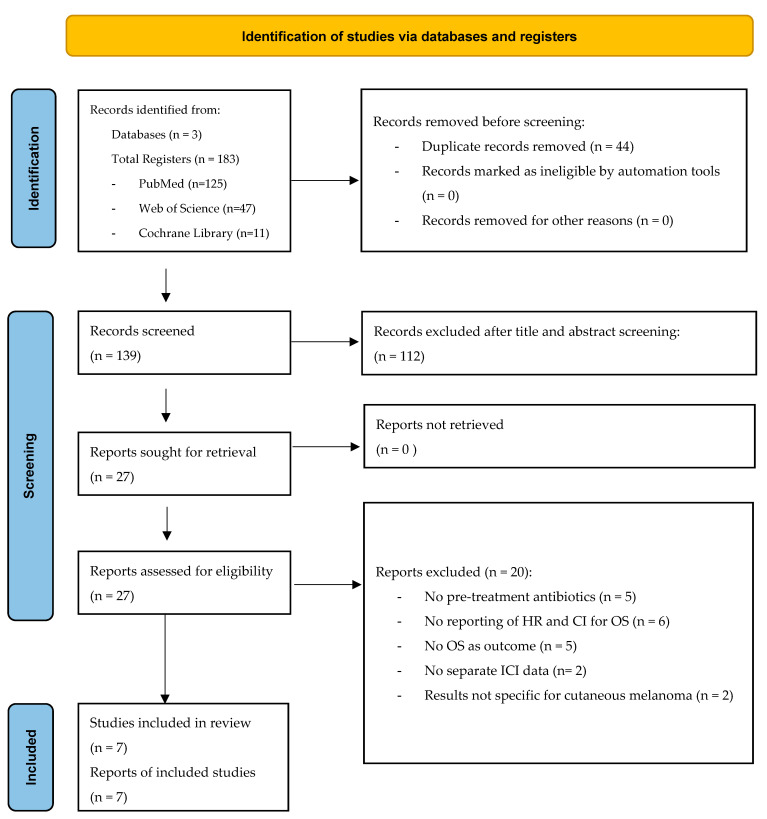
PRISMA flowchart of the present systematic review and meta-analysis.

In the present systematic review and meta-analysis [21,22,23,24,25,26,27], the Preferred Reporting Items for Systematic Reviews and Meta-Analyses (PRISMA) guidelines were followed Figure 1 [28]. The PRISMA checklist for this review is provided in Table S1.

### 2.2. Search Strategies

We performed a systematic search of PubMed, Web of Science, and Cochrane Library databases, and the references of the retrieved articles were examined. Studies published between 1 January 2010 and 1 May 2025 that assessed the association between the use of antibiotics and OS in patients with CM who had received ICI were included. The most recent search of the databases listed above was conducted on 2 May 2025. The last search in the databases listed above was undertaken on 2 May 2025. The following combinations of search keywords in the abstract and title were used: (nivolumab OR pembrolizumab OR ipilimumab OR immune checkpoint inhibitor OR PD-1 inhibitor OR CTLA-4 inhibitor) AND (antibiotic OR anti-infectious OR antibiotics) AND (melanoma). We also screened the reference lists of papers selected for this study.

### 2.3. Selection of Studies and Data Extraction

Following identification of potential studies, two independent reviewers (T.G. and S.S.W.F.), selected the included articles in three phases. In screening phase 1, the reviewers evaluated the titles and abstracts based on the eligibility criteria. In phase 2, the reviewers reviewed the full texts and selected the articles according to the same criteria as in screening phase 1. Subsequently, the reviewers verified all the information obtained concerning critical inclusion and exclusion criteria. In cases of disagreement, a third reviewer (L.S. or N.A.R.) was consulted. Inclusion criteria were as follows: Studies including at least 30 patients with advanced (stage III/IV) CM, first-line ICI, antibiotics up to 90 days prior to the initiation of ICI, hazard ratios (HR) including the 95% confidence intervals (CI) for OS based on multivariable analysis. Papers reporting antibiotic use under ICI, abstracts, posters, reviews, case reports, and patients with uveal melanoma were excluded from further analysis. Data were collected on the characteristics of the studies, including authorship, study design, and year of publication and country, ICI modality, timing of antibiotics prior ICI, number of patients with and without (controls) antibiotics, results, and conclusion. The primary outcome was the assessment of OS, defined as the time from initiation of first ICI for CM to death or last date of follow-up [21,22,23,24,25,26,27].

### 2.4. Individual Assessment of Study Quality

The Newcastle–Ottawa Scale (NOS) is a frequently used tool for evaluating the quality of non-randomized studies, such as cohort and case-control studies, included in systematic reviews and meta-analyses. The NOS assesses the quality of a study based on three main aspects: selection of study groups, comparability of the groups, and assessment of the outcomes or exposures [29].

### 2.5. Data Synthesis and Statistical Analysis

All analyses were performed using the meta and metafor packages in R and RStudio (version 4.4.3). We synthesized log-transformed hazard ratios (log-HRs) and their standard errors from each study using both fixed- and random-effects models. All tests were two-sided, and *p* < 0.05 was considered statistically significant unless otherwise specified.

### 2.6. Primary Meta-Analysis and Forest Plot

We synthesized the log-transformed hazard ratios (log-HRs) from seven observational cohorts of melanoma patients to estimate the overall effect of antibiotic exposure prior to ICI therapy on survival. Using the meta package in R, we fitted both fixed-effect and random-effects models (REML estimator) and displayed each study’s log-HR (square) with its 95% confidence interval (horizontal line) alongside a pooled summary diamond. Between-study heterogeneity was evaluated with Cochran’s Q, I^2^, and τ^2^. Because I^2^ exceeded 50%, we additionally calculated a 95% prediction interval, which was superimposed on the forest plot, to convey the plausible range of true effects in a new, comparable study in accordance with PRISMA recommendations.

### 2.7. Sensitivity Analyses

To verify the stability of our findings, we first conducted a leave-one-out analysis, sequentially omitting each study and re-estimating the pooled HR and heterogeneity statistics; this allowed us to observe the minimum and maximum pooled effects and the corresponding shifts in I^2^. We then produced a Baujat plot, which visualizes each study’s contribution to total heterogeneity on the *x*-axis against its influence on the pooled estimate on the *y*-axis, thereby identifying any outliers or high-leverage studies. Finally, we applied the Duval and Tweedie trim-and-fill procedure to assess and correct for potential publication bias by estimating the number of missing studies and recalculating the adjusted pooled HR.

### 2.8. Subgroup Analyses

Recognizing that study-level factors might drive heterogeneity, we stratified the data according to ICI regimen (monotherapy versus PD-1 + CTLA-4 combination), overall sample size (categorized at the median), and geographic origin (France versus other countries). For each category, we refitted the random-effects model and generated subgroup-specific forest plots, reporting pooled HRs alongside updated heterogeneity metrics (I^2^ and τ^2^) to compare effect magnitudes and consistency across subgroups.

### 2.9. Meta-Regression

To formally test whether particular study characteristics explained between-study variability, we fitted mixed-effects meta-regression models using meta’s metareg() function. We examined four moderators in separate models: the midpoint of the antibiotic-to-ICI window (continuous), ICI regimen (binary: monotherapy versus combination), total sample size (continuous), and study country (binary: France versus other). For each, we reported the residual heterogeneity (τ^2^, I^2^), the proportion of variance explained by the moderator (R^2^), the QM statistic evaluating the moderator’s overall effect, and the estimated regression coefficient (β) with its 95% CI and *p*-value.

### 2.10. Publication Bias Assessment

We assessed potential small-study effects by constructing a manually labeled funnel plot of log-HRs against their standard errors, marking each study by its index number. Egger’s regression test evaluated funnel asymmetry, with *p* < 0.10 considered indicative of possible bias. We complemented this with the trim-and-fill adjustment to estimate and impute any missing studies and to derive an adjusted pooled HR.

## 3. Results

### 3.1. Extracted Data and Study Characteristics

As shown in Table 1, seven observational studies (n = 5213 patients; 1074 with prior antibiotic exposure, 4139 without) were included in the primary meta-analysis (Chorti et al. [21]; Cren et al. [22]; Elkrief et al. [23]; Gaucher et al. [24]; Mohiuddin et al. [25]; Poizeau et al. [26]; Vihinen et al. [27]). Individual study HRs for overall survival ranged from 1.01 to 2.60, with wider confidence intervals observed in smaller cohorts (e.g., Elkrief et al. [23]). Not all studies uniformly reported the indications for antibiotic use, the specific agents prescribed, or the routes of administration. Overall, antibiotics were most often given for skin and soft tissue infections, including postoperative wound infections, cellulitis, and erysipelas, as well as for respiratory and urinary tract infections. The most commonly used agents were penicillins, cephalosporins, and quinolones, administered either orally or intravenously.

### 3.2. NOS Assessment

The seven non-randomized studies showed a generally low risk of bias, with four (Elkrief et al. [23]; Gaucher et al. [24]; Mohiuddin et al. [25]; Vihinen et al. [27]) achieving perfect NOS scores (9/9). Chorti et al. (2024) scored 8/9 due to unclear follow-up completeness, while Cren et al. [22] and Poizeau et al. [26] scored 7/9, each lacking detail on exposure ascertainment or follow-up adequacy. All cohorts were appropriately selected and balanced for key confounders, and outcome assessment was blinded or independent in six studies. Follow-up duration was sufficient in all but Poizeau et al. [26], and completeness was well-documented except in Chorti et al. [21] and Cren et al. [22]. Overall, the high selection and comparability ratings bolster confidence in these findings, with only minor reporting gaps in three studies (Table 2).

### 3.3. Primary Meta-Analysis

**Figure 2:**
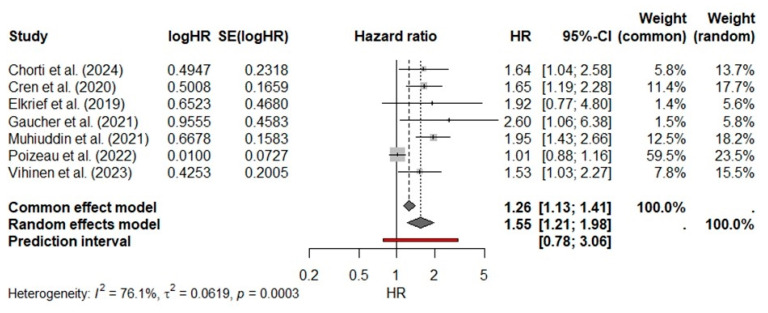
Forest plot of seven studies [21,22,23,24,25,26,27] assessing the impact of antibiotic exposure prior to immune-checkpoint inhibitor therapy on overall survival in melanoma. Columns show each study’s log-HR, standard error, HR with 95% CI, and relative weights under fixed-effect (“common”) and random-effects models. Squares represent individual study HRs (size proportional to weight) with horizontal lines as 95% CIs. The black diamond denotes the pooled HR under the random-effects model (HR 1.55; 95% CI 1.21–1.98), and the gray diamond shows the fixed-effect estimate (HR 1.26; 95% CI 1.13–1.41). The red horizontal bar beneath the diamonds is the 95% prediction interval [0.78, 3.06], indicating the expected range of true HRs in a new study given the observed heterogeneity (I^2^ = 76.1%, τ^2^ = 0.0619, *p* = 0.0003).

Under a fixed-effect model, prior antibiotic exposure was significantly associated with poorer survival (pooled HR 1.26, 95% CI 1.13–1.41; *p* < 0.001). However, heterogeneity was high (Q = 25.05, df = 6, *p* < 0.001; I^2^ = 76.1%), justifying a random-effects approach. The random-effects model yielded a similarly significant association (pooled HR 1.55, 95% CI 1.21–1.98; *p* = 0.0003), indicating that patients who received antibiotics in the 30–90 days preceding ICI had a ~55% higher hazard of death compared with those who did not. At first glance, these findings underscore a consistent adverse impact of recent antibiotic use on ICI efficacy in melanoma (Figure 2). However, heterogeneity remained high. The 95% prediction interval ranged from 0.78 to 3.06, indicating that in some future cohorts, the HR could be as low as 0.78 or as high as 3.06.

### 3.4. Sensitivity Analysis

**Figure 3:**
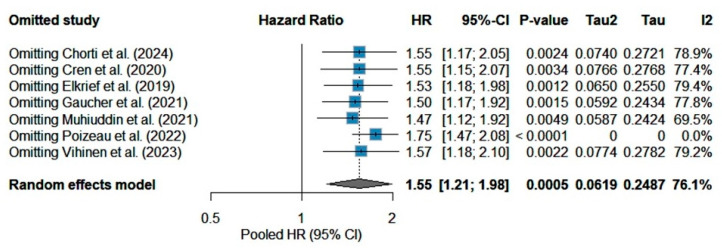
The leave-one-out plot shows how the overall random-effects estimate and heterogeneity change when each study is omitted in turn [21,22,23,24,25,26,27]. The pooled hazard ratio remains elevated and highly significant (HR ≈ 1.50–1.75; all *p* < 0.01) no matter which single study is dropped. This indicates that the adverse association of prior antibiotics with overall survival is not driven by any one study. However, when Poizeau et al. [26] is omitted, I^2^ falls to 0% and τ^2^ to 0, suggesting that this study, whose HR is very close to null [1.01 (0.88–1.17)], contributes most of the between-study variability.

**Figure 4:**
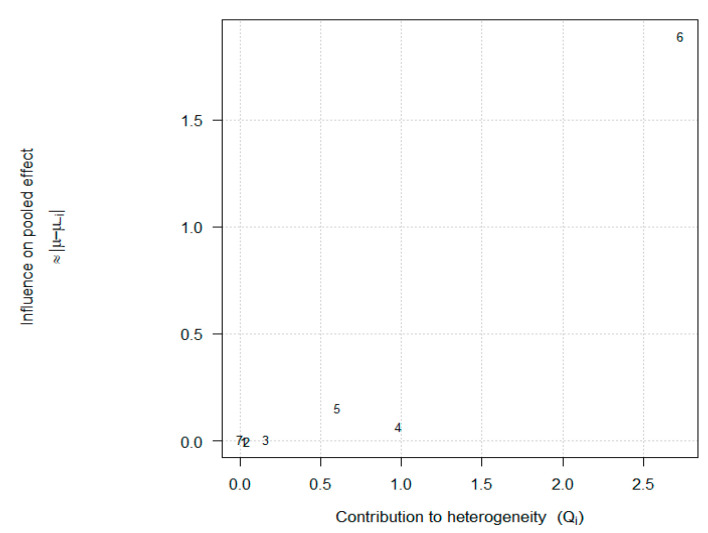
Baujat-style influence plot of seven studies (1 = Chorti, 2 = Cren, 3 = Elkrief, 4 = Gaucher, 5 = Mohiuddin, 6 = Poizeau, 7 = Vihinen) [21,22,23,24,25,26,27]. The *x*-axis (Q_i_) quantifies each study’s contribution to the overall heterogeneity statistic, while the *y*-axis (|μ–μ(–_i_)|) shows how much the pooled fixed-effect estimate shifts when that study is omitted. Points are labeled by first-author surname. Studies in the top-right quadrant (study# 6) drive both heterogeneity and influence the pooled hazard ratio most strongly.

In the leave-one-out analysis, pooled HRs remained between 1.50 and 1.75 (all *p* < 0.01) when each study was excluded in turn (Figure 3). Omitting Poizeau et al. [26] produced the largest increase to approximately 1.75 and reduced I^2^ to 0%, underscoring its role as the primary source of heterogeneity. To identify which studies most strongly drive the overall effect and the observed heterogeneity, we generated a Baujat-style influence plot (Figure 4). On the *x*-axis, Poizeau et al. [26] made the largest contribution to the χ^2^ heterogeneity (Q_i_), reflecting its neutral hazard ratio in contrast to the other cohorts. On the *y*-axis, the same study also produces the greatest shift in the pooled fixed-effect estimate when omitted (|μ–μ(–_i_)|), confirming it as the principal source of inconsistency. Gaucher et al. [24] likewise contributed to heterogeneity, albeit to a lesser extent, whereas smaller or more directionally consistent studies (e.g., Elkrief, Chorti) lay near the origin, indicating minimal influence. These diagnostics corroborate our leave-one-out findings and highlight Poizeau et al. [26] as the key outlier study warranting further investigation of its design and exposure definition. The trim-and-fill procedure imputed one potentially missing study on the left side of the funnel and yielded an adjusted HR of 1.46 (95% CI 1.08–1.97), demonstrating modest attenuation.

### 3.5. Meta-Regression Subgroup Analyses

To explore whether specific study-level characteristics could account for the observed heterogeneity, we fitted four separate mixed-effects meta-regression models, each adding one moderator at a time while retaining a residual random-effects component (REML estimator). When the ICI regimen (monotherapy vs. combination therapy) was entered as a categorical moderator, the variance between studies dropped markedly: τ^2^ fell from 0.0985 in the unadjusted model to 0.0422, and I^2^ declined from 76.1% to 54.0%. The model attributed 31.9% of the between-study variance to differences in regimen (R^2^ = 31.9%). The estimated coefficient for monotherapy (relative to combination therapy) was (0.3522 on the log-HR scale) equivalent to a 30% lower hazard (HR = e^−0.3522^ ≈ 0.70), but this did not reach statistical significance (95% CI −0.8060 to 0.1017; *p* = 0.1283; QM = 2.31), indicating that, despite its substantial explanatory power for heterogeneity, ICI regimen type alone did not fully account for effect-size differences.

In the model testing the midpoint of the antibiotic-to-ICI window (in days) as a continuous predictor, τ^2^ decreased more modestly to 0.0546 and I^2^ to 59.7%, with the window explaining 11.8% of the total variance (R^2^ = 11.8%). The regression slope of −0.0077 per day implied that each additional day between antibiotic exposure and ICI initiation was associated with an approximate 0.8% reduction in the HR (HR multiplier ≈ 0.992), but again, this effect was not statistically significant (95% CI −0.0206 to 0.0052; *p* = 0.244; QM = 1.35), suggesting that timing prior ICI initiation alone does not strongly predict outcomes.

When the total study size was assessed as a continuous moderator, virtually no heterogeneity was resolved: τ^2^ remained at 0.087, I^2^ at 74.0%, and only 3.1% of the variance was explained (R^2^ = 3.1%). The coefficient of −0.00015 per additional patient hinted at a negligible trend toward smaller effect estimates in larger cohorts, but this was non-significant (95% CI −0.00038 to 0.00008; *p* = 0.21).

Finally, grouping by country (France vs. all others) yielded τ^2^ = 0.0468 and I^2^ = 54.5%, with the country accounting for 24.3% of between-study variance (R^2^ = 24.3%). The France indicator coefficient (β = −0.2596; SE = 0.2301) corresponded to a non-significant relative hazard reduction of about 23% (HR ≈ 0.77; 95% CI 0.52–1.11; *p* = 0.259; QM = 1.27), indicating no meaningful difference in the antibiotic effect between French and non-French studies.

### 3.6. Publication Bias

**Figure 5:**
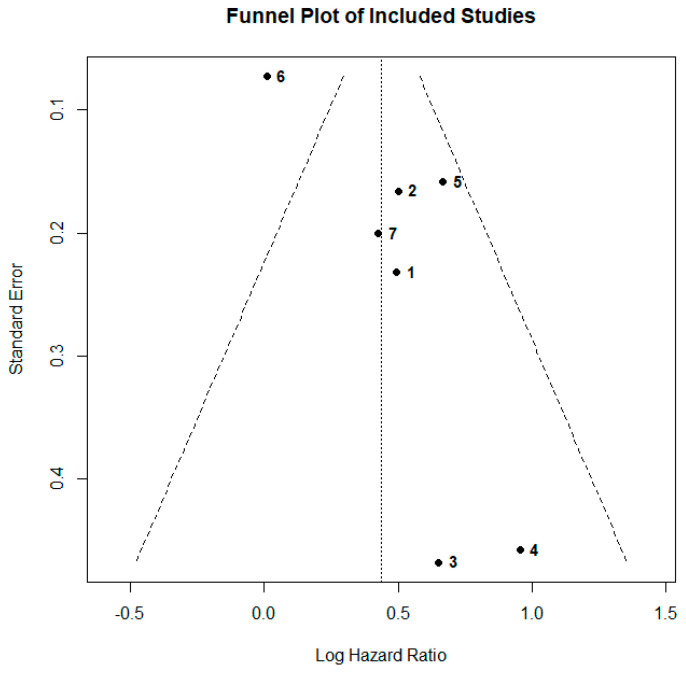
Funnel plot of seven studies’ log-hazard ratios versus their standard errors. The dashed lines show the pseudo–95% confidence limits around the pooled effect (vertical dotted line). Each point is labeled by study number (1 = Chorti, 2 = Cren, 3 = Elkrief, 4 = Gaucher, 5 = Muhiuddin, 6 = Poizeau, 7 = Vihinen) [21,22,23,24,25,26,27]. Studies 3 and 4 lie in the lower-right quadrant, driving the observed asymmetry.

Visual inspection of the funnel plot (Figure 5) revealed that the two smallest and most imprecise studies, Elkrief et al. [23] (study 3) and Gaucher et al. [24] (study 4), both lie to the lower-right of the funnel, with elevated log-HRs and large standard errors and no counterparts on the left, creating marked asymmetry. Egger’s regression suggested significant small-study effects (intercept = 2.87, *p* = 0.024); however, with only seven studies, Egger’s test has low power and may produce misleading results when fewer than ten studies are included. Nevertheless, these findings underscored the need for sensitivity analyses (e.g., trim-and-fill) and careful scrutiny of study-level differences when interpreting the pooled hazard ratio.

## 4. Discussion

Our meta-analysis of seven observational cohorts demonstrates a consistent association between prior antibiotic exposure and diminished overall survival in melanoma patients treated with ICI. Both fixed-effect and random-effects models yielded significantly elevated hazard ratios—26% and 55% increases in mortality risk, respectively—underscoring a robust signal across diverse settings. The wide 95% prediction interval (0.78–3.06) cautions that in some future cohorts, the effect may be negligible, whereas in others, it could be markedly detrimental, reflecting substantial clinical and methodological heterogeneity.

Exploration of heterogeneity revealed that the ICI regimen accounted for the largest share of variability. Combination PD-1 + CTLA-4 studies coalesced around a higher pooled HR (1.91) with moderate heterogeneity, whereas monotherapy cohorts were more variable (HR = 1.34; I^2^ = 79%). Our leave-one-out and Baujat analyses flagged specific monotherapy studies, particularly Poizeau et al. [26], as outliers driving inconsistency. This suggests that the impact of antibiotics may differ by ICI mechanism, potentially reflecting regimen-specific interactions with the gut microbiome or immune milieu. Meta-regression supported these observations: regimen type explained nearly one-third of between-study variance, while antibiotic-to-ICI interval, sample size, and geography contributed minimally.

The modest attenuation of heterogeneity when adjusting for antibiotic-to-ICI interval (R^2^ = 11.8%) implies that timing may influence outcomes to a limited extent but does not fully account for effect-size differences. Similarly, study size and geographic location explained only 3.1% and 24.3% of the variance, respectively, indicating that unmeasured factors—such as antibiotic class, dosing, underlying infection severity, or patient comorbidities—likely contribute importantly to between-study differences. The latter details were not consistently available in the studies selected.

The presence of small-study effects, suggested by funnel asymmetry and a significant Egger intercept, underscores how publication bias might inflate the pooled HR: smaller studies reporting strong associations are more likely to be published than those with null findings, potentially overestimating the true effect. Indeed, our trim-and-fill adjustment attenuated the HR from 1.55 to 1.46, highlighting that the observed association may be partly driven by selective reporting and reinforcing the need for large, prospective cohorts to obtain unbiased estimates.

Antibiotics administered before ICI therapy can profoundly alter the gut microbiome, depleting commensals, such as *Akkermansia* and *Faecalibacterium*, that promote anti-tumor immunity and enhance T cell activation. Preclinical models confirm that dysbiosis reduces dendritic-cell maturation and blunts PD-1 inhibitor efficacy, while early clinical data (e.g., NCT05102773) link richer baseline microbial diversity to higher response rates and more pronounced immune-related adverse events [30,31,32,33,34]. Clinically, these insights argue for the implementation of antibiotic stewardship in oncology settings: development of evidence-based guidelines, routine multidisciplinary review with infectious disease specialists before initiating broad-spectrum antibiotics, and integration of decision-support tools to minimize unnecessary microbiome disruption.

Our analysis is constrained by several factors. First, retrospective designs cannot fully adjust for confounding by indication: patients receiving antibiotics may have had more severe infections, poorer performance status, or other comorbidities that independently worsen survival. Second, exposure windows varied (30–90 days) and lacked granularity on antibiotic class, dose, and route. Third, with only seven studies and fewer per subgroup, statistical power for meta-regression and bias tests was limited. Finally, unmeasured factors such as baseline microbiome composition and supportive care practices likely influence outcomes and warrant incorporation in future research. Moving forward, prospective, microbiome-integrated trials will be critical to confirm causality, identify microbial biomarkers predictive of ICI response, and enable personalized immunotherapy strategies in melanoma.

## 5. Conclusions

Prior-ICI antibiotic use is consistently associated with worse survival among melanoma patients receiving ICI, with the greatest detriment seen under combination PD-1 + CTLA-4 regimens. Although the ICI regimen explains a substantial portion of between-study variability, residual heterogeneity and small-study effects indicate that publication bias and unmeasured clinical factors may influence the observed associations. Our findings underscore the need for judicious antibiotic stewardship in oncology, particularly the development of evidence-based prescribing guidelines co-designed by oncologists and infectious disease specialists, and prospective studies that rigorously characterize antibiotic timing, patient comorbidities, and longitudinal microbiome changes. Going forward, integrating comprehensive microbiome profiling into clinical trials will be critical to validate causality, discover predictive microbial biomarkers, and personalize ICI therapy in melanoma. This call to action emphasizes precision-medicine approaches as the pathway to optimize outcomes and advance the field.

## Figures and Tables

**Table 1:** Basic characteristics of studies (n = 7) included in the primary meta-analysis of melanoma patients who underwent treatment with immune checkpoint inhibitors (ICI).

Study	Year	Location	Study Design	Pabt No/Yes	ICI Type	ATB Prior ICI (Days)	HR for OS 95% CI
Chorti [21]	2024	Germany	observational, multi-center	428/41	PD1 PD1+CTLA-4	60	1.64 1.04–2.58
Cren [22]	2020	France	observational, large database	1514/71	CTLA-4	60	1.65 1.19–2.28
Elkrief [23]	2019	Canada	observational, oligo-center	64/10	PD1 PD1+CTLA-4	30	1.92 0.76–4.76
Gaucher [24]	2021	France	observational, single-center	94/16	PD1 PD1+CTLA-4 CTLA-4	30–60	2.60 1.06–6.39
Mohiuddin [25]	2021	USA	observational, large database	454/114	PD1 PD1+CTLA-4 CTLA-4	90	1.95 1.43–2.66
Poizeau [26]	2022	France	observational, large database	1856/749	PD1	90	1.01 0.88–1.17
Vihinen [27]	2023	Finland	observational, multi-center	151/71	PD1	30–90	1.53 1.03–2.26

pABT = prior antibiotics; PD1 = programmed cell death protein; CTLA-4 = cytotoxic T lymphocyte-associated protein 4; HR = hazard ratio; 95% CI = 95% confidence interval.

**Table 2:** The overall quality and risk of bias in the identified studies were evaluated using the NOS (NEWCASTLE—OTTAWA Quality Asessment Scale) for non-randomized controlled trials selected in the present systematic review according to PRISMA.

Study	Selection				Comparability	Outcome		
	S1	S2	S3	S4	C	O1	O2	O3
Chorti et al. [21]	★	★	★	★	★★	★	★	
Cren et al. [22]	★	★		★	★★	★	★	
Elkrief et al. [23]	★	★	★	★	★★	★	★	★
Gaucher et al. [24]	★	★	★	★	★★	★	★	★
Mohiuddin et al. [25]	★	★	★	★	★★	★	★	★
Poizeau et al. [26]	★	★		★	★★	★		★
Vihinen et al. [7]	★	★	★	★	★★	★	★	★

A study can be awarded a maximum of one star for each numbered item within the selection and outcome categories. A maximum of two stars can be given for comparability: S1: representativeness of the exposed cohort; S2: selection of the non-exposed cohort; S3: ascertainment of exposure; S4: demonstration that outcome of interest was not present at start of study; C: comparability of cohorts on the basis of the design or analysis; O1: assessment of outcome, O2: was follow-up long enough for outcomes to occur? O3: adequacy of follow-up of cohorts.

## Data Availability

All data relevant to the study are included in the article.

## Supplementary Materials

The following supporting information can be downloaded at: https://www.mdpi.com/article/10.3390/cancers17111872/s1, Table S1: PRISMA 2020 Checklist [35].
